# The Role of e-NOS in Chronic Cholestasis-Induced Liver and Renal Injury in Rats: The Effect of N-Acetyl Cysteine

**DOI:** 10.1155/2014/564949

**Published:** 2014-11-09

**Authors:** Yusuf Gunay, Semsi Altaner, Nergiz Ekmen

**Affiliations:** ^1^Department of Surgery, Atakent Hospital, Acibadem University, Halkali, 34678 Istanbul, Turkey; ^2^Department of Pathology, Baskent University Istanbul Hospital, Altunizade, 34567 Istanbul, Turkey; ^3^Department of Gastroenterology, Florence Nightingale Hospital, Bilim University, Sisli, 34321 Istanbul, Turkey

## Abstract

*Introduction.* The role of chronic cholestasis (CC) in liver injury and fibrosis remains unclear. The aims of this study were to define the role of endothelial nitric oxide synthase (e-NOS) in CC and the protective effect of N-acetyl-L-cysteine (NAC) in liver and kidney injury. *Materials and Methods.* Group A (sham group); Group B (CBDL); and Group C (CBDL + NAC). Group C received daily dosage of NAC (100 mg/kg) intraperitoneally for up to 4 weeks. *Results.* The rate of bridging fibrosis was higher (100% versus 20%, *P* = .025), but the intensity of e-NOS in liver was lower in rats that received NAC (1.3 versus 2.7, *P* = .046). The necrotic area in the kidneys among rats that received NAC was lower at week 4 (48% versus 57%; *P* < .001). The numbers of e-NOS stained cells in kidney were similar in sham group and the two groups with CBDL. *Discussion.* NAC reduced the stimulus for liver fibrosis in this rat model of CC and attenuated liver and kidney injury. Our study showed that e-NOS expression increased in liver tissue of rats with CC and that this was reversed by NAC. Treatment with NAC might restore e-NOS protein expression and prevent liver injury in CC.

## 1. Introduction

Experiments have shown that common bile duct ligation (CBDL) results in chronic cholestasis (CC) and consequent liver inflammation, fibrosis, and cirrhosis [1, 2]. The role of CC in liver injury and fibrosis remains unclear. Possible mechanisms include accumulation of hydrophobic bile acids, free radicals, and decreased antioxidative activity [3, 4]. Moreover, CC increases production of nitric oxide (NO) that reacts with free oxygen radicals and leads to cellular damage and apoptosis [5]. Several studies have shown that multiple organ injury occurs in CC [6–8]. CC with liver dysfunction is frequently accompanied by kidney injury [9–12], which could be caused by endotoxemia, vasoactive mediators, and free radicals [13–15]. In addition, renal injury associated with liver diseases could occur by direct tubular toxicity or nephron obstruction [16].

N-Acetyl-L-cysteine (NAC) is a precursor of glutathione and acts as a direct scavenging agent [17]. Studies show that NAC increases the glutathione and antioxidant pools in hepatic cells and thus increases the resistance of hepatic cell membranes [18]. In addition to its antioxidant effects, NAC exerts anti-inflammatory properties by inhibiting cytokines and adhesion molecules and increasing production of endothelial nitric oxide synthase (e-NOS) [19, 20]. Previous studies have shown that NAC improved renal function in ischemia-/reperfusion-induced acute kidney injury. NAC protection of organs primarily involves the scavenging of oxygen-free radicals [21] and modulates e-NOS expression [22]. The results suggest that increased e-NOS expression by NAC may play a crucial role in protection of organs from injuries.

Little is known about how e-NOS is linked to liver and renal injury in CC or how it protects CC patients from liver and kidney injury. The aims of this study were to establish a rat model of CC, define the role of e-NOS in CC, and learn how NAC exerts protective effects in liver and kidney injury via e-NOS.

## 2. Materials and Methods

The studies followed recognized guidelines for the use of experimental animals, were approved by the ethical committee of Bogazici University (Istanbul, Turkey) (number 2013-06-04), and were conducted in accordance with the Ministry of Food, Agriculture and Livestock of Turkey and international laws (Guide for Care and Use of Laboratory Animals, 1996). Female Wistar rats (200–250 g) were housed at constant temperature with 12 h dark/light cycles. Animals were allowed access to standard rat chow and water. All rats were anaesthetized with intramuscular ketamine (50 mg/kg) and xylazine (7 mg/kg). Sixty-one rats were randomized into three groups: Group A (sham group, 5 rats); Group B (CBDL, 28 rats); and Group C (CBDL + NAC, 28 rats). Group B and Group C both had 7 rats at weeks 1 and 2. At week 3, Group B had 6 rats (one rat died) and Group C had 7 rats. At week 4, one rat in Group C had died; therefore, both Groups B and C had 6 rats at the time of sacrifice.

### 2.1. Surgery

Experimental CC was induced by CBDL through a midline laparotomy. The common bile duct was doubly ligated with a 4–0 silk suture and was transected between the ligatures. The abdominal wall was closed with interrupted silk sutures. Group B received normal saline (NS) and Group C received NAC (100 mg/kg) intraperitoneally for their first treatment. They subsequently received 100 mg/kg daily dosage of NS or NAC intraperitoneally for up to 4 weeks. Rats were harvested at 1, 2, 3, and 4 weeks after CBDL. After the laparotomy, blood was collected weekly for biochemical analysis. At week 4 the rats were sacrificed and livers and kidneys were harvested for histopathological studies.

### 2.2. Histopathology

Tissue samples were fixed in 10% formalin for 24 h, rinsed with tap water for 24 h, and dehydrated using a graded alcohol series. Tissues were made transparent in xylene and embedded in paraffin. Sections (5 *μ*m) were stained with hematoxylin-eosin (H&E), periodic acid Schiff (PAS), and Masson's trichrome stain. Changes in liver histology were classified using the Scheuer classification as follows [23]:

 0: no changes;
 1: portal inflammation with bile duct damage with or without florid duct lesions;
 2: ductular reaction (with periportal fibrosis often present);
 3: bridging fibrosis;
 4: biliary cirrhosis.

Renal hematoxylin and eosin stained sections were evaluated for proximal tubule necrosis and apoptosis, endothelial cell apoptosis, simplification, juxtaglomerular apparatus hypertrophy, and cell number by an experienced renal pathologist (blinded to sample identity). Tissue damage was scored using a tubule damage score that measured the percentage of damaged tubules (those exhibiting loss of brush border, dilation, cast formation, and cell lysis) [24] and the presence of periodic acid Schiff-positive granules in renal tubules: 1, <25% tubules; 2, 25–50% tubules; 3, 50–75% tubules, and 4, >75% tubules [25].

### 2.3. Immunohistochemistry

Expression of e-NOS was detected in kidney and liver using a rabbit polyclonal antibody (BioGen, RB-9279-R7, USA) and the streptavidin-biotin peroxidase technique [4]. The procedure was performed under identical conditions for all sections. Paraffin sections (5 *μ*m) were dewaxed in xylene. The sections were rehydrated, rinsed in deionized water, and subjected to 2 N HCl solution for 20 min to enhance antigen retrieval. Endogenous peroxidase activity was inhibited in 3% H_2_0_2_ in methanol for 10 min. The sections were washed in phosphate buffered saline (PBS) three times (5 min each) and preincubated in 1.5% normal goat serum in PBS for 20 min at room temperature in a humidified chamber.

The sections were incubated overnight at 4°C with e-NOS antibody (2 *μ*g/mL diluted in PBS with 1.5% NGS). For the negative controls, PBS was used in place of the primary antibody. Sections were incubated with biotinylated goat anti-rabbit IgG (1 *μ*g/mL diluted in PBS with 1.5% NGS) for 30 min at room temperature in a humidified chamber and then with streptavidin horseradish peroxidase conjugate (ready-to-use) for 30 min. Finally, the sections were counterstained with hematoxylin, rinsed in deionized water, and mounted on glass slides with clear mounting solution (ready-to-use). The sections were examined and photographed using a light microscope (Bx-51). Immunohistochemical e-NOS staining intensity in the kidney and liver sections from all groups was evaluated semiquantitatively by two independent histologists (blinded). The intensity of e-NOS expression was scored as none (−), low (+), moderate (++), and strong (+++).

### 2.4. Serum Biochemistry

Serum samples were stored at −80°C. Serum creatinine (SCr) and alanine aminotransferase (ALT) levels were determined by spectrophotometric methods using an autoanalyzer (RxL-Max, Siemens, Munich, Germany).

### 2.5. Statistical Analysis

Data are reported as arithmetic means (M) and standard deviations (SD) of 5–7 animals in each treatment group. Statistical analysis included Student's *t*-test and Mann-Whitney nonparametric *U* test. *P* < .05 was considered statistically significant (SSPS, Inc., Chicago, IL).

## 3. Results

### 3.1. CBDL and Liver Fibrosis

**Figure 1:**
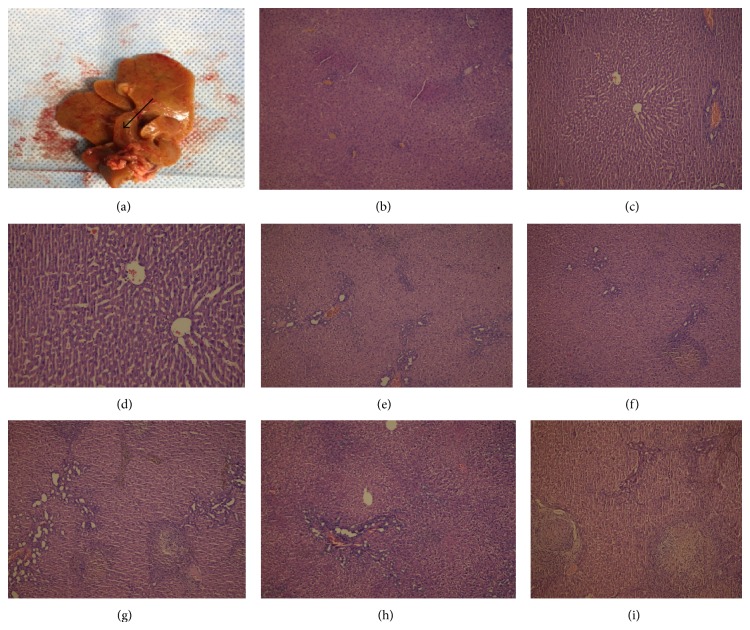
(a) Dilated common bile duct in rats (4 weeks after CBDL). (b) (Group A), (c) (Group B), and (d) (Group C): histological changes among groups (1 week after the CBDL). Minimal dilatation in the portal region, a few mononuclear cells, and ductal proliferation in portal regions are seen (H&E, ×200). (e) (Group B) and (f) (Group C): more intense inflammation and more ductile proliferation were observed (2 weeks after CBDL) (H&E, ×200). (g) (Group B): severe bridging fibrosis with enlargement of the portal region (H&E, ×200). (h) (Group C): no bridging fibrosis with enlargement of the portal region (H&E, ×200). (i) (Group B).

CBDL for 1, 2, 3, or 4 weeks was used to induce CC among rats in Group B (CBDL only) and Group C (CBDL + NAC). The effect of CC on liver and kidney was evaluated by biochemical analysis and histological examination of each week. After 4 weeks, the CBDL rats showed a dilated common bile duct (Figure 1(a)). While no changes were noted in the sham group (Group A), the rats in Groups B and C developed liver inflammation and bile duct injury that was detected after the first week. Their portal tracts were minimally enlarged with an inflammatory infiltration that included lymphocytes, neutrophils, and hepatocellular ballooning. In addition, ductile proliferation was observed in Groups B and C where histological changes were similar among rats (Figures 1(b), 1(c), and 1(d)). At week 2, portal inflammation included more leukocytes with a few plasmocytes and the bile duct injury was more intense than that observed initially. Periportal fibrosis was observed in both Groups B and C. While less periportal fibrosis was observed in rats receiving NAC (Group C), this difference was not statistically significant (66% versus 50%, *P* = .89, Figures 1(e) and 1(f)).

**Figure 2:**
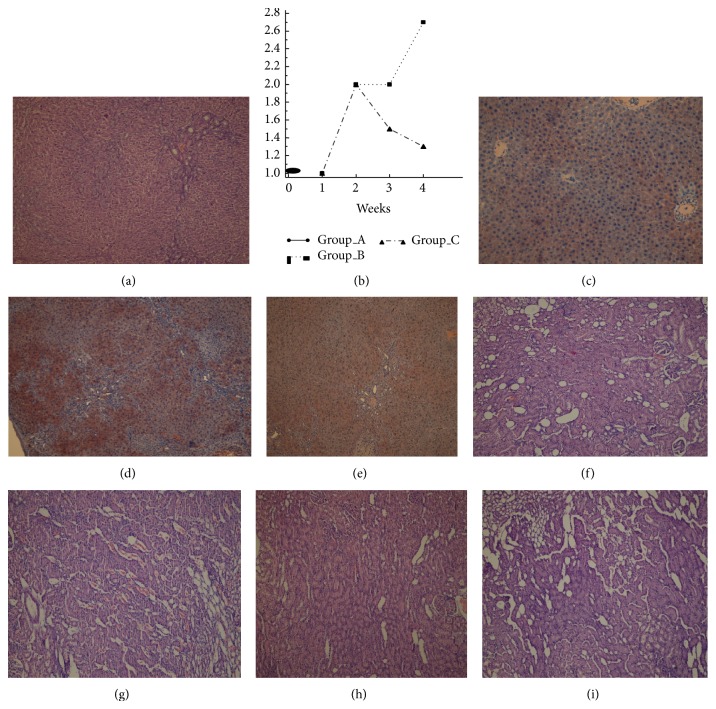
(a) (Group C): enlargement of the portal region and bridging fibrosis present in Group B. Enlargement of the portal region was present in Group C, but no bridging fibrosis was present (H&E, ×200). (b) Rat liver e-NOS expression for each week: no staining (0), low (1), moderate (2), and strong (3). (c) Weak cytoplasmic expression of e-NOS in hepatocytes (eNOS, ×200). (d) (Group B) and (e) (Group C): hepatocyte cytoplasmic expression of e-NOS was weaker in Group C than in Group B (eNOS, ×200). (f) (Group B) and (g) (Group C): the necrotic area in Group B was more intense than in Group C (H&E, ×200). (h) (Group B) and (i) (Group C): the necrotic area in Group B was more intense than in Group C (H&E, ×200).

CBDL in rats was associated with increases in periportal fibrosis, bile duct proliferation, and bridging fibrosis in Group B as confirmed by Masson's trichrome staining of liver tissue at week 3 (Figure 1(g)). Although Group C exhibited inflammation, bile duct proliferation, and periportal fibrosis, no bridging fibrosis was observed (Figure 1(h)). The extent of liver bridging fibrosis was significantly lower in rats receiving NAC (80% versus 0, *P* = .045). At week 4, bridging fibrosis was observed among all rats in Group B but only in one of five rats in group C (100% versus 20%, *P* = .025). This suggests that NAC reduced the stimulus for fibrosis in this rat model of CC and attenuated progression of liver injury (Figures 1(i) and 2(a)).

The expression of e-NOS in rat liver was scored for each group every week (Figure 2(b)). The mean number of cells that stained positively for e-NOS was low in the sham group (Group A; Figure 2(c)). In Groups B and C, the number of cells that stained positively for e-NOS was similar to that found for Group A at week 1. No significant differences were found between the Groups B and C at weeks 1 and 2. The mean intensity of e-NOS staining increased in Groups B and C after the second week. At week 3, the mean e-NOS expression was lower in rats receiving NAC, but this difference was not statistically significant (2 versus 1.5, *P* = .55). At week 4, Group C expressed e-NOS at levels that were significantly lower than those found for Group B (1.3 versus 2.7, *P* = .046; Figures 2(d) and 2(e)).

### 3.2. NAC Administration Prevents Renal Injury after CBDL

Histological examination of kidneys of rats in sham group (Group A) did not reveal any pathology, whereas rats in both CBDL groups showed low levels of tubular epithelial injury and neutrophil infiltration at week 1. Small foci of coagulation necrosis in the renal cortex were found on both PAS and H&E stained sections at week 1 and the necrotic areas in kidneys were significantly smaller in the rats that received NAC (4% versus 11%, *P* < .001, Table 1 and Figures 2(f) and 2(g)). At week 2, tubular epithelial injury was observed in the kidney cortex, with small foci of coagulation necrosis among rats in Groups B and C. Dilated tubules and an increase in proteins and cell casts were observed in distal tubules and most prominently in collecting ducts in the cortex (Figures 2(h) and 2(i)). The necrotic areas observed in kidneys among rats that received NAC were significantly smaller than rats in Group B at week 2 (21% versus 26%, *P* < .001, Table 1).

**Figure 3:**
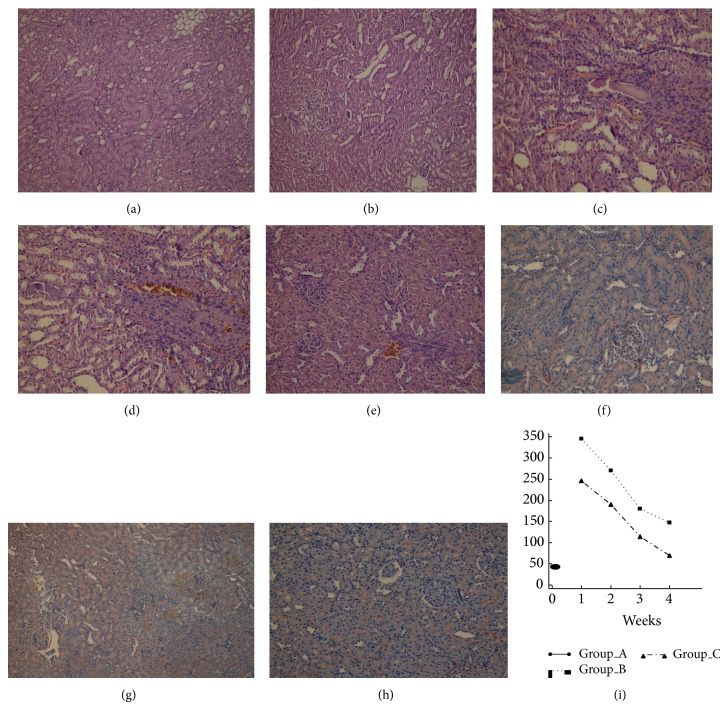
(a) (Group B) and (b) (Group C): the necrotic area was more intense in Group B than in Group C (H&E, ×200). (c) (Group B): chronic pyelonephritis in Group B with increased fibrosis and tubular hyalinization in tubules (H&E, ×200). (d) (Group B) and (e) (Group C): in Group B, there was increased intertubular inflammation and fibrosis and tubular atrophy (chronic pyelonephritis), but no chronic pyelonephritis was observed in Group C (H&E, ×200). (f) (Group A): weak e-NOS expression in proximal and distal tubular cytoplasm (eNOS, ×200). (g) (Group B) and (h) (Group C): proximal and distal tubular cytoplasmic weak positivity of e-NOS expression was present in Group B and C, but no staining was present in glomeruli (eNOS, ×200). (i) ALT level in three groups for each week postprocedure.

At week 3, cortical tubules exhibited focal, cytoplasmic vacuolization, dilated lumina, focal granular casts, and mild interstitial edema. Proximal tubules showed significantly higher numbers of necrotic cells in the CBDL rats. The necrotic area in kidneys among rats that received NAC was significantly smaller than rats in Group B at week 3 (48% versus 57%; *P* < .001; Table 1 and Figures 3(a) and 3(b)). Focal pyelonephritis was observed in kidneys of the Group B rats, whereas it was not observed in the rats that received NAC (Group C; Figure 3(c)). Moreover, among Group B rats, all the tubules were dilated, and the tubular cells were atrophied. These histological changes were less serious in the rats that received NAC. Endothelial apoptosis of peritubular capillaries was visible in H&E sections as linear apoptotic bodies corresponding to interstitial capillary endothelium in group B rats. The necrotic area in the kidneys among rats that received NAC (Group C) was significantly lower at week 4 (54% versus 62%; *P* < .001; Table 1 and Figures 3(d) and 3(e)).

Expression of e-NOS was primarily examined in distal and collecting ducts and within glomeruli and distal and collecting duct tubules. Expression of e-NOS in the kidney of rats in sham group (Group A) showed little eNOS staining around the distal and collecting duct tubules and no e-NOS staining in glomeruli (Figure 3(f)). The numbers of e-NOS stained cells in collecting and distal ducts were similar in the two groups with CBDL (Groups B and C) and were not larger than those observed in Group A. NAC had no effect on e-NOS expression in kidney at week 4 in Groups B and C, which had similar e-NOS staining intensity in glomeruli (no staining), collecting ducts (little staining), and distal ducts (little staining). The intensity of e-NOS staining was not affected by NAC in rats with CBDL (Figures 3(g) and 3(h)).

### 3.3. Biochemical Analysis

ALT was analyzed every week and the mean level was higher in CBDL groups than in the sham group (Figure 3(i)). ALT levels were lower in Group C than in Group B at 4 weeks (68 ± 30 U/L versus 146 ± 63 U/L, *P* = .03). Kidney function was evaluated by serum creatinine at week 4. The mean creatinine concentration in the CBDL groups was significantly higher than that in sham group (2.3 ± 0.3 mg/dL in Group B and 1.6 ± 0.5 mg/dL in Group C versus 0.34 ± 0.1 mg/dL in Group A; *P* < .001 and *P* = .001). The mean concentration of creatinine was significantly lower in Group C than in Group B (*P* = .006).

## 4. Discussion

A chronic cholestatic rat model was used to examine the role of e-NOS and the protective effects of NAC in kidney and liver injury. NAC treatment of CBDL rats prevented liver and renal injury. Our study showed that e-NOS expression increased in liver tissue of rats with CC and that this was reversed by NAC treatment. Expression of e-NOS in renal tissue did not change in rats with CC and its expression was not affected by NAC.

Several studies have demonstrated the effect of CC in liver and kidney injury [1, 2, 9–12], and several factors may play a role [3, 4, 13–15]. Systemic oxidative stress, increased hepatic lipid peroxidation, hepatocellular mitochondrial dysfunction, release of proinflammatory cytokines from the liver after liver injury, circulating bile acids, endotoxins, circulating immune complexes, decreased glutathione production, and excessive production of NO occur in CC [26–29]. A potential role of NO in the development of liver fibrosis has been demonstrated [30, 31]. NO can react with superoxide anion and reactive oxygen species and cause formation of peroxynitrite anion, increased lipid peroxidation, and tissue injury in kidney and liver [32–35]. However, there is little information in the literature regarding the role of e-NOS in NO production and liver and kidney injury in CC liver diseases.

The use of NAC in our study was justified because it decreases oxidative stress and exerts anti-inflammatory and immunomodulatory effects [21, 36–39]. CBDL has been reported to decrease antioxidant defenses and liver concentrations of glutathione (GSH) and increase free radical formation [40]. Studies have suggested a role for free radicals in the modulation of hepatic fibrogenesis, either directly or through lipid peroxidation [41, 42]. The antifibrotic effects of NAC have been demonstrated in experimental models [43, 44]. Moreover, the activity of serum enzymes (ALT) was reduced in animals that underwent CBDL and received NAC, which demonstrates the hepatoprotective effect of this drug [45–48]. The interaction of NAC and e-NOS protein expression was studied previously [49]. NAC acts as a superoxide scavenger, capable of tripling e-NOS expression and increasing NO bioavailability [50, 51]. However, it is not known whether NAC acts in the e-NOS expression pathway to attenuate liver and kidney injury in CC.

We investigated the role of e-NOS in liver and kidney injury and the effect of NAC in e-NOS expression and its protection against the progression of liver and kidney injury in CC. The CC + NAC group (Group C) showed reduced hepatic injury and lower amounts of e-NOS in liver tissue than the CC group without NAC (Group B). Treatment with NAC resulted in reduced activation of e-NOS and accumulation of collagen in liver. There was a significant decrease in collagen staining after treatment with NAC once a day for 4 consecutive weeks after CBDL. The increase in the concentration of ALT after CBDL was less in rats that received NAC suggesting reduced hepatic inflammation. Overall, this study showed that NAC prevents liver injury caused by CC liver diseases. NAC reduced e-NOS expression in liver and attenuated inflammation, leading to a significant reduction in cellular damage, hepatocyte injury, and fibrosis. This is the first report to demonstrate the role of e-NOS in liver injury in CC and the novel effect of NAC acting via e-NOS in attenuating liver injury in CC. Treatment with NAC might restore e-NOS protein expression and prevent liver injury in CC.

This study shows that NAC ameliorates the deleterious effects of CBDL in liver and kidneys, a protection that appears to be related to potentiation of e-NOS. These findings suggest that NAC supplementation in conjunction with other treatments might be beneficial in patients with chronic cholestatic disorders to protect kidneys and liver; however, further experimental and randomized clinical studies are needed. This study has some limitations. First, we did not examine the effect of NAC in the presence of other free radicals that might cause liver fibrosis and renal injury. It is possible that NAC may affect other free radical agents and diminish renal and liver injury via other pathways. Second, the precise role of NAC in preventing renal injury was not identified in this study because e-NOS expression in renal tissue was not affected by either CC or NAC treatment.

In summary, a model of kidney and liver injury after CC liver disease characterized by renal tubular necrosis, endothelial cell apoptosis, polymorphonuclear leukocyte infiltration, and liver fibrosis was demonstrated. It will be useful to delineate the role of e-NOS in CC and to evaluate the potential for NAC therapy. Continued investigation of the mechanism whereby e-NOS contributes to liver fibrosis may lead to novel approaches for treating liver injury in patients with CC.

## Figures and Tables

**Table 1:** Mean necrotic areas in kidneys in groups.

Time	Group B (mean ± SD %)	Group C (mean ± SD %)	*P*	95% CI
Week 1	11 ± 1.5	4 ± 0.8	<0.001	3.2–12.5
Week 2	26 ± 1	21 ± 1.4	<0.001	19.1–28.4
Week 3	57 ± 2.2	48 ± 2.6	<0.001	45.3–60.8
Week 4	62 ± 0.5	54 ± 2.1	<0.001	51.9–62.5
